# Spatial localisation in autism: evidence for differences in early cortical visual processing

**DOI:** 10.1186/2040-2392-4-4

**Published:** 2013-02-19

**Authors:** Keziah Latham, Susana TL Chung, Peter M Allen, Teresa Tavassoli, Simon Baron-Cohen

**Affiliations:** 1 Department of Vision & Hearing Sciences, Anglia Ruskin University, Cambridge, UK; 2 Vision and Eye Research Unit, Anglia Ruskin University, Cambridge, UK; 3 School of Optometry, University of California, Berkeley, CA, USA; 4 Department of Psychiatry, Autism Research Centre, Cambridge University, Cambridge, UK; 5 Department of Psychiatry, Seaver Autism Centre, Mount Sinai School of Medicine, New York, NY, USA

**Keywords:** Autism spectrum conditions, Spatial vision, Vernier acuity, Hyperacuity, Psychophysics, Visual processing

## Abstract

**Background:**

Vision in people with autism spectrum conditions (ASC) is reported to be different from people without ASC, but the neural level at which the differences begin to occur is not yet known. Here we examine two variants of a vernier acuity task to determine if differences are evident in early visual processing.

**Findings:**

Abutting and separated vernier acuity was assessed in 16 people with ASC and 14 matched controls. In controls, abutting and separated thresholds were unrelated (*r* = 0.13, *p* = 0.65), suggesting thresholds are determined by two separate mechanisms. In contrast, the abutting and separated thresholds of ASC observers were strongly correlated (*r* = 0.88, *p* < 0.0001), with separated thresholds tending towards being superior to those of controls [t(28) = −2.46, *p* = 0.02].

**Conclusions:**

The findings suggest the mechanisms employed by ASC observers in separated vernier tasks are different to those of controls. This psychophysical evidence suggests that visual differences in ASC may begin at an early cortical stage of visual processing.

## Findings

Where in the visual processing system do differences in vision begin to arise for people with autistic spectrum conditions (ASC)? People with ASC appear to have normal responses to basic visual tasks [1], but difficulties with complex face recognition tasks and enhanced attention to local visual information over global in tasks such as visual search [2,3]. To understand the nature and location of differences in visual processing in ASC, Simmons et al. [3] state: “A really basic characterization of the visual processing capabilities of people with ASC would be extremely useful, even if all it could do was say with certainty ‘nothing is wrong here’”.

Starting with basic visual processing, visual acuity (or the minimum recognisable acuity) is limited by foveal cone spacing [4] and is similar in people with ASC and controls [1]. Moving methodically up the visual pathway, the next logical visual function to examine is the spatial localisation of two features, or the minimum discriminable acuity. Localisation thresholds can be more precise than cone spacing and are limited in the primary visual cortex [5-8].

Here, we assess spatial localisation using a vernier acuity paradigm across two conditions that are thought to be mediated by different neural mechanisms: (1) *abutting* line vernier targets, processed by contrast-dependent spatial filters encompassing both stimulus elements [5,6]; (2) line vernier targets *separated* by 10 arc min. For lines separated by more than 4 arc min [5,6], vernier thresholds are contrast-independent, the mechanism underlying performance involving position identification of each stimulus element using a local sign process [9], followed by a collator mechanism capable of comparing the responses to the two individual stimulus elements [6-8]. Our hypothesis, based on earlier evidence, is that spatial localisation in ASC and control observers will be similar, with differences arising at higher (attentional) levels of visual processing.

## Methods

### Procedure

**Figure 1:**
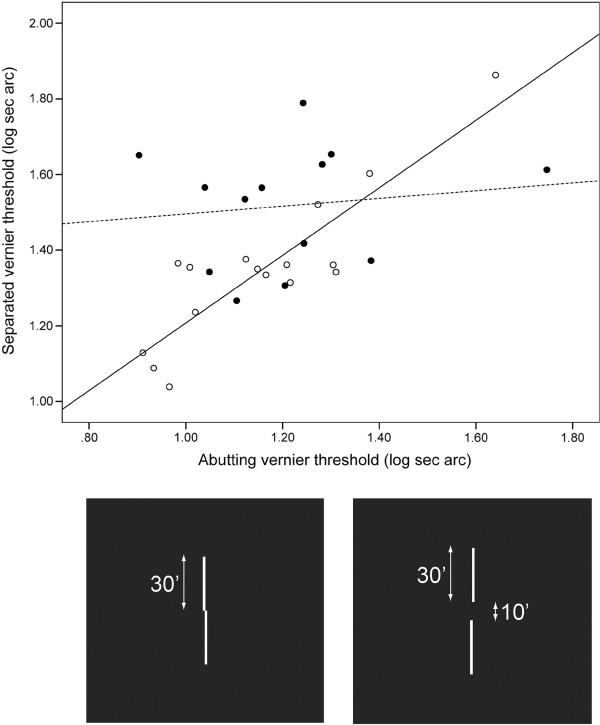
**Abutting and separated vernier thresholds (log sec arc).** Stimulus configurations are shown in insets **a** and **b** respectively. *Filled points*: controls; *open points*: ASC. *Dotted line*: best fit to controls [separated threshold = 1.40 + (0.10 × abutting threshold)]; *solid line*: best fit to ASC [separated threshold = 0.31 + (0.89 × abutting threshold)].

Two-line vernier thresholds were assessed using two long (30 arc min at 8 m), thin (18.9 arc sec at 8 m), bright (luminance 300 cdm^-2^) white lines presented vertically on a dark (luminance <3.4 cdm^-2^) background in a darkened room on an iMac running OS10.6.3 under the control of PsychoPy v1.64.00 [10]. There were two conditions: with the lines abutting and with the lines separated vertically by a gap of 10 arc min (shown in inset to Figure 1). For each condition, the upper test line was drawn with a horizontal offset with respect to the lower reference line. Seven offset positions were tested, where the upper line was 1, 2 or 3 steps to the right or left of the lower line, along with a zero-offset condition (the two lines were aligned vertically). The step size was adjusted for each observer during the practice blocks (see below). Each offset was tested 15 times, in a random order, within a block of trials. The observers’ task was to use a two-alternative forced-choice paradigm (right or left) to indicate, via a keyboard, the direction of the upper test line with respect to the lower reference line. Testing was performed binocularly with observers wearing their habitual spectacle correction that resulted in a visual acuity of at least 0.00logMAR.

The position of the test stimulus presentation was jittered between trials to prevent the observers from making judgements based on the absolute position of the test line with respect to other cues such as the edge of the monitor display and was presented until a response was made. Response times were recorded and no feedback was given. Initially a practice session was undertaken with three trials per block to check that step sizes covered the range from approximately 0-100% rightward responses and to familiarise the observer with the task and response keys. This was repeated if necessary to confirm step size requirements. The 105 trials in each block took approximately 2–5 min to complete. Four blocks were completed for each observer: half in the order *AGGA* and half in the order *GAAG* (where *A* is the abutting condition and *G* the condition separated by a gap).

For each block of trials, the number of rightward responses was tallied for each offset position. A cumulative Gaussian function was used to fit the data, from which we derived two important parameters: (1) the offset from the zero-offset condition (the two lines were perfectly aligned) that corresponded to 50%-rightward response, representing the response bias, and (2) the offset between the two lines to change the rightward response on the psychometric function from 50 to 84%, representing the vernier threshold. This definition of vernier threshold is equivalent to 1 SD of the cumulative Gaussian function that was fitted to the observer’s responses.

The authors conducting the experiments and analysing the data were blind to whether participants were in the ASC or control groups until after data had been collected and analysed.

### Participants

Participants were included if they met the following inclusion criteria: aged over 18 years, an Intelligence Quotient [IQ: Wechsler Abbreviated Scale of Intelligence (WASI)] of at least 90 so as to exclude those with ‘low average’ IQ or below [11], no self-reported cognitive co-morbidities, corrected habitual binocular visual acuity at least 0.00 logMAR and no manifest strabismus as assessed by optometric screening. In addition, participants with ASC had a clinical diagnosis based on DSM-IV criteria and an Autism Spectrum Quotient (AQ) score of at least 26 [12]. Controls had an AQ score of less than 22. Ethical approval for the study was obtained from Anglia Ruskin University, and informed consent was obtained from all participants.

Data were collected for 17 people with ASC and 16 controls. Data from two controls were excluded from analysis as their AQ scores were >26 and from one person with ASC as their WASI IQ was <90. Data are therefore presented from 16 people with ASC and 14 controls as shown in Table 1. The two groups did not differ in terms of age, gender or IQ (all *p* > 0.05), but as expected did differ on Autism Spectrum Quotient (AQ) scores (*p* < 0.0001).

**Table 1:** Sample characteristics

	**ASC (*n *= 16)**	**Controls (*n *= 14)**	**Two-tailed independent *t*-test (Levene > 0.05; equal variances assumed)**
	**Mean ± SD (range)**	**Mean ± SD (range)**	
Age	34.4±9.8 years (20–54 years)	38.1 ± 6.3 years (26–48 years)	*t* = 1.19, df28, *p* = 0.24
Gender	9 female, 7 male	6 female, 8 male	Pearson chi-square 0.54, *p* = 0.46
WASI	120.5 ± 10.9 (99–135)	115.5 ± 9.4 (100–130)	*t* = 1.30, df26, p = 0.20
AQ	40.7 ± 4.6 (29–48)	12.4 ± 4.3 (4–21)	*t* =16.6, df28, *p* < 0.0001
VA	−0.15 ± 0.09 logMAR (0.00 - -0.28)	−0.16 ± 0.10 logMAR (0.00 - -0.30)	*t* = 0.27 df27 *p* = 0.79

## Results

There was no difference in response bias for abutting stimuli [ASC −2.5 ± 4.7, control −2.2 ± 5.7; t(28)-1.15, *p* = 0.88] or for separated stimuli [ASC −3.2 ± 8.8, control +3.4 ± 9.0; t(28)-2.01, *p* = 0.06].

Mean vernier thresholds for each group and condition are shown in Table 2. Thresholds for the control group were in accordance with previously published data for similar parameters [5]. There was no significant difference in thresholds between ASC and control observers for abutting vernier stimuli [t(28) = −0.63, *p* = 0.54; Cohen’s *d* −0.25]. There was a significant difference between groups for separated vernier thresholds [t(28) = −2.52, *p* = 0.018, Bonferroni corrected; Cohen’s *d* = −0.96, effect size *r* =−0.43], with ASC observers’ thresholds being better than controls’.

**Table 2:** Group-mean vernier thresholds (± SD) in log sec arc, taken as the geometric mean of two blocks of responses for each participant

	**ASC**	**Control**
Abutting	1.16 ± 0.20	1.21 ± 0.20
Separated	1.35 ± 0.20	1.52 ± 0.15

However, of greater interest than the mean difference between the groups is the relationship between abutting and separated vernier thresholds for the two groups as shown in Figure 1. In controls (filled points), the abutting and separated thresholds are unrelated (Pearson *r* = 0.13, p = 0.65). In ASC (open points), the abutting and separated thresholds rise in proportion to one another (Pearson *r* = 0.88, *p* < 0.0001).

It would be expected that abutting and separated vernier thresholds would be unrelated, since in typical controls they have been shown to be determined by two separate mechanisms. As previously outlined, abutting thresholds are determined by contrast-dependent spatial filters encompassing both elements [5,6], whilst separated thresholds are determined by a two-step process of local sign designation followed by comparison of features [6-8]. The results of the control observers are consistent with these findings.

The finding of a strong correlation between abutting and separated vernier thresholds in the ASC observers suggests that their thresholds for the two conditions may be determined by the same mechanism. People with ASC may have spatial filters that act over greater separations than in control observers, such that they do not need to switch to a comparison mechanism at a 10 arc min separation. However, it is not clear how such larger spatial filters would have the capacity to retain a high accuracy of vernier judgement. Future work to test this hypothesis might include an exploration of the influence of contrast [5], reverse polarity elements [6] or spatial frequency masks [13] on the separated vernier thresholds of observers with ASC.

The finding of differences in ASC in the mechanisms used to determine vernier thresholds is not consistent with visual differences in autism being solely due to changes in higher level attention rather than early sensory processing [2,14], nor are the findings consistent with the suggestion that visual thresholds for tasks with two stages of processing, such as the separated vernier task, are poorer in ASC because of atypical cortical lateral connectivity [15]. Neuroanatomical studies suggest high local connectivity between smaller minicolumns in the autistic primary visual cortex [16] that might be implicated in differences in threshold mechanisms in people with ASC, and atypical lateral connectivity has also been suggested to explain psychophysical findings of better contrast detection in the presence of lateral masks in ASC [17]. Enhanced performance in ASC has been shown for visual simultaneity thresholds [18], a localisation task in the temporal domain, which is similar to the task reported here in the spatial domain.

In conclusion, we show that vernier thresholds for abutting and separated vernier stimuli are not related in controls but are strongly correlated in ASC, suggesting that people with ASC employ different mechanisms than controls in processing separated vernier stimuli. These findings provide psychophysical evidence of a difference in visual processing in ASC at an early stage of cortical visual processing and demonstrate that visual changes in ASC are not restricted to higher level perceptual processes.

## Abbreviations

ASC: Autism spectrum conditions; AQ: Autism Spectrum Quotient; IQ: Intelligence Quotient; WASI: Wechsler Abbreviated Scale of Intelligence

## Competing interests

The authors declare that they have no competing interests.

## Authors’ contributions

KL conceived of the study, participated in the data collection, analysed the data and led preparation of the manuscript. SC conceived of the study, programmed the stimuli and analysed the data. PA and TT participated in the design of the study and data collection. SBC participated in the design of study. All authors helped to draft and revise the manuscript and have read and approved the final manuscript.
